# The relationship between VEGF-460(T>C) polymorphism and cancer risk: A systematic review and meta-analysis based on 46 reports

**DOI:** 10.1097/MD.0000000000034089

**Published:** 2023-06-30

**Authors:** Haoran Qin, Qiang Xiao, Yufen Xie, Dan Li, Xiaozhou Long, Taiping Li, Siqing Yi, Yiqin Liu, Jian Chen, Foyan Xu

**Affiliations:** a General Surgery Department, First Affiliated Hospital of Nanchang University, Nanchang, China; b Department of Mammary Diseases, Zhuhai Hospital of Integrated Chinese and Western Medicine, Zhuhai, China; c General Surgery Department, Zhuhai Hospital of integrated Traditional Chinese and Western Medicine, Guangdong, China.

**Keywords:** meta-analysis, neoplasms, polymorphism, single nucleotide, VEGF-460(T>C)

## Abstract

Extensive studies on the link between single nucleotide polymorphisms (SNPs) in vascular endothelial growth factor (VEGF) and various malignancy risks produced conflicting results, notably for VEGF-460(T/C). To evaluate this correlation more comprehensively and accurately, we perform a meta-analysis.

**Methods:**

Through retrieving 5 databases (Web of Science (WoS), Embase, Pubmed, Wanfang database (Wangfang), and China National Knowledge Infrastructure (CNKI)) and applying hand search, citation search, and gray literature search, 44 papers included 46 reports were enrolled. To evaluate the relationship between VEGF-460 and cancer risk, we pooled odds ratios (ORs) and 95% confidence intervals (CIs).

**Results:**

Our results indicated that the VEGF-460 polymorphism is not related to malignancy susceptibility (dominant model, OR = 0.98, 95% CI = 0.87–1.09; recessive model, OR = 0.95, 95% CI = 0.82–1.10; heterozygous model, OR = 0.99, 95% CI = 0.90–1.10; homozygous model, OR = 0.92, 95% CI = 0.76–1.10; additive model, OR = 0.98, 95% CI = 0.90–1.07). While, in subgroup analysis, this SNP may reduce the risk of hepatocellular carcinoma.

**Conclusion:**

this meta-analysis indicated that VEGF-460 was irrelevant to overall malignancy risk, but it might be a protective factor for hepatocellular carcinoma.

## 1. Introduction

Worldwide, cancer imposes a massive burden on human health, second only to ischemic heart disease and stroke,^[1]^ and is a critical barrier to increasing life expectancy.^[2]^ According to statistics, 19.3 million newly diagnosed cancer cases and approximately 10 million cancer deaths occurred worldwide in 2020.^[3]^ The World Health Organization (WHO) prediction model stated that cancer would replace ischemic heart disease as the major death cause over the next 4 decades.^[1]^ Efforts on researching the pathogenesis of malignancies to administer effective interventions in carcinogenesis and progression are fundamental to cancer control worldwide. Molecular epidemiological findings have demonstrated that genetic factors, especially single nucleotide polymorphisms (SNPs), are vital in oncogenesis and progression.^[4,5]^

The human VEGF gene is localized on chromosome 6p21.3 and spans over 16 kb, with a coding region consisting of 8 exons and 7 introns.^[6,7]^ These exons selectively splice micro-RNA in different combinations to generate multiple coding products. Five homologous members, VEGF-A (commonly known as VEGF), VEGF-B, VEGF-C, VEGF-D, and placental growth factor, comprise the VEGF family.^[8]^ VEGF receptors R1 and R2 were discovered successively as VEGF signaling receptors.^[9]^ VEGF-R1 is a tyrosine kinase VEGF receptor with high affinity and displays weak ligand-dependent tyrosine autophosphorylation.^[10]^ VEGF-R2 is the main signaling receptor associated with mitogenesis and permeability of vascular endothelial cells and thus is thought to be involved in tumorigenesis and progression.^[11]^ Ligand bound to VEGF-R2 triggers autophosphorylation of intracellular tyrosine residues.^[12]^ VEGF binds specifically the VEGF receptor (VEGFR) in its extracellular domain, contributing to the formation of homo- or heterodimers of the VEGFR. Tyrosine residues 1214 (Y1214) and 1175 (Y1175) on VEGF-R2 are highly autophosphorylated in response to VEGF and Y1175 is essential for VEGF-dependent cell proliferation via the phospholipase Cγ (PLCγ)/PKC/mitogen-activated protein kinase pathway in cultured endothelial cells.^[13]^ The dimerized VEGFR stimulates intracellular tyrosine kinases and induces autophosphorylation of tyrosine residues, which in turn activates a series of downstream signal transduction pathways, including PI3K/Akt and mitogen-activated protein kinases.^[14]^ Thus, it exerts various biological functions, including promoting endothelial cell mitosis, inhibiting endothelial cell apoptosis, vasodilation, increasing vascular permeability.^[9,15]^

As a crucial process of oncogenesis and development, angiogenesis is necessary for primary tumor cells’ growth, invasion, and metastasis.^[16]^ Moreover, VEGF overexpression has been reported in many malignancies.^[17]^ Over 30 SNPs were previously reported in the VEGF gene; parts have been demonstrated to modulate VEGF expression levels^[18]^ and may be associated with cancer susceptibility. Numerous institutions have performed extensive studies to demonstrate the relationship between SNPs in VEGF and cancer, especially for VEGF-460(T/C).^[19–78]^ However, different and even contradictory results were obtained. Several investigators have conducted meta-analyses to evaluate this link comprehensively.^[79–92]^ Their meta-analysis was almost only for single cancer with small sample sizes. As far as we know, only one meta-analysis of VEGF-460 polymorphisms and overall cancer risk has been published,^[79]^ but it was published earlier and included less studies. Therefore, we performed a meta-analysis with sufficient literature to investigate the strength of the link between VEGF-460 and overall malignancy.

## 2. Materials and methods

The data in this meta-analysis was accessed from literature published or from other sources that did not directly involve patients. Consequently, this work did not require ethics committee approval and informed consent. This meta completes registration on the PROSPERO platform with the registration ID CRD42021241267.

### 2.1. Literature strategy

We accessed related publications in 5 databases: WoS, Embase, Pubmed, Wanfang, and NCKI, until February 2022, without language restrictions. The complete search strategy is provided in Table S1, Supplemental Digital Content, http://links.lww.com/MD/J156 (See Table S1, Supplemental Digital Content, http://links.lww.com/MD/J156, which demonstrates the specific search strategies.). We also searched the reference of eligible studies to obtain as much of the available literature as possible. To reduce the bias risk possible, gray literature, such as these, and conference papers, was also considered. Two authors completed the above search independently and screened according to the following criteria.

#### 2.1.1. Inclusion criteria.

Studies related to VEGF-460(T/C) polymorphism and malignancy (no restriction on cancer type). Case-control studies: pathologically diagnosed malignant tumors in case groups. Studies with access to complete genotype frequencies.

#### 2.1.2. Exclusion criteria.

Repeated literature. Non-human trials. For multiple articles with data that overlap in whole or in part, we select articles published more recently or with more extensive data volumes. *P* value of the Hardy–Weinberg equilibrium (HWE) ≤ 0.05.

If 2 authors reach different inclusion studies, discussion with a third author is essential. The 3 authors must agree on one point of view.

### 2.2. Data extraction

Two authors separately extracted information below: author and year, nation, ethnicity, cancer type, control sources, genotype frequency, and *P* value of the HWE in control groups. If HWE were not reported in the original research, the chi-square test would be used to assess it; *P* > .05 was considered that the genotype distribution was conformed to HWE in the control group. If a dispute arises between 2 authors, a discussion with a third author is required to resolve the disagreement.

### 2.3. Quality assessment

Since studies we included were case-control trials, quality assessment was performed with the Newcastle–Ottawa scale (NOS).^[93]^ The NOS for case-control studies contained 3 aspects (selection, comparability, and exposure) included 8 items. Scoring criteria are provided for each item, except for comparability, which can get 2 stars, other items passed with 1 star. A total of 9 stars, 6 stars, and above are regarded as high quality. Two authors were evaluated separately and discussed with a third author.

### 2.4. Statistical analysis

#### 2.4.1. Quantitative synthesis.

We combined ORs with 95% CIs to estimate the link between VEGF-460 polymorphism and malignancy risk in 5 genetic models (dominant, recessive, heterozygous, homozygous, and additive models). The primary outcome was the effect of VEGF-460 polymorphism on cancer susceptibility; secondary outcomes were the relationship between the 2 in terms of ethnicity, cancer type, and control source.

#### 2.4.2. Heterogeneity analysis.

We used Cochran’s Q test to ascertain heterogeneity among the primary studies.^[94]^ P_Heterogeneity_ < 0.10 (not 0.05) represents heterogeneity as Cochran’s Q test has low statistical strength.^[95]^ We also used *I*^2^ values to quantitatively assess heterogeneity, with *I*^2^ values of 20%, 50%, and 75% indicating low, medium, and high heterogeneity, respectively.^[96]^ If there was no heterogeneity among the original researches, the ORs and 95% CIs were combined using fixed-effects models; otherwise, random-effects models were applied. Exploration of the sources of heterogeneity was conducted with meta-regression and subgroup analysis; the *P* value of meta-regression less than .05 represents the heterogeneity.

#### 2.4.3. Publication bias.

We used the contour-enhanced funnel plots, Begg’s, and Egger’s tests for the estimation of publication bias.^[97]^ Moreover, asymmetry in the funnel plots or *P* values below .05 in either test suggested publication bias.

#### 2.4.4. Sensitivity analysis.

The leave-one-out method, which removes individual studies on a rotating basis to recombine ORs, was performed to determine the stability of the outcomes. This work was considered steady if there were no drastic changes or reversals in the recombined results.

#### 2.4.5. Software.

The above analysis is implemented on stata15.0.

## 3. Results

### 3.1. Characteristics of the studies

**Figure 1.**
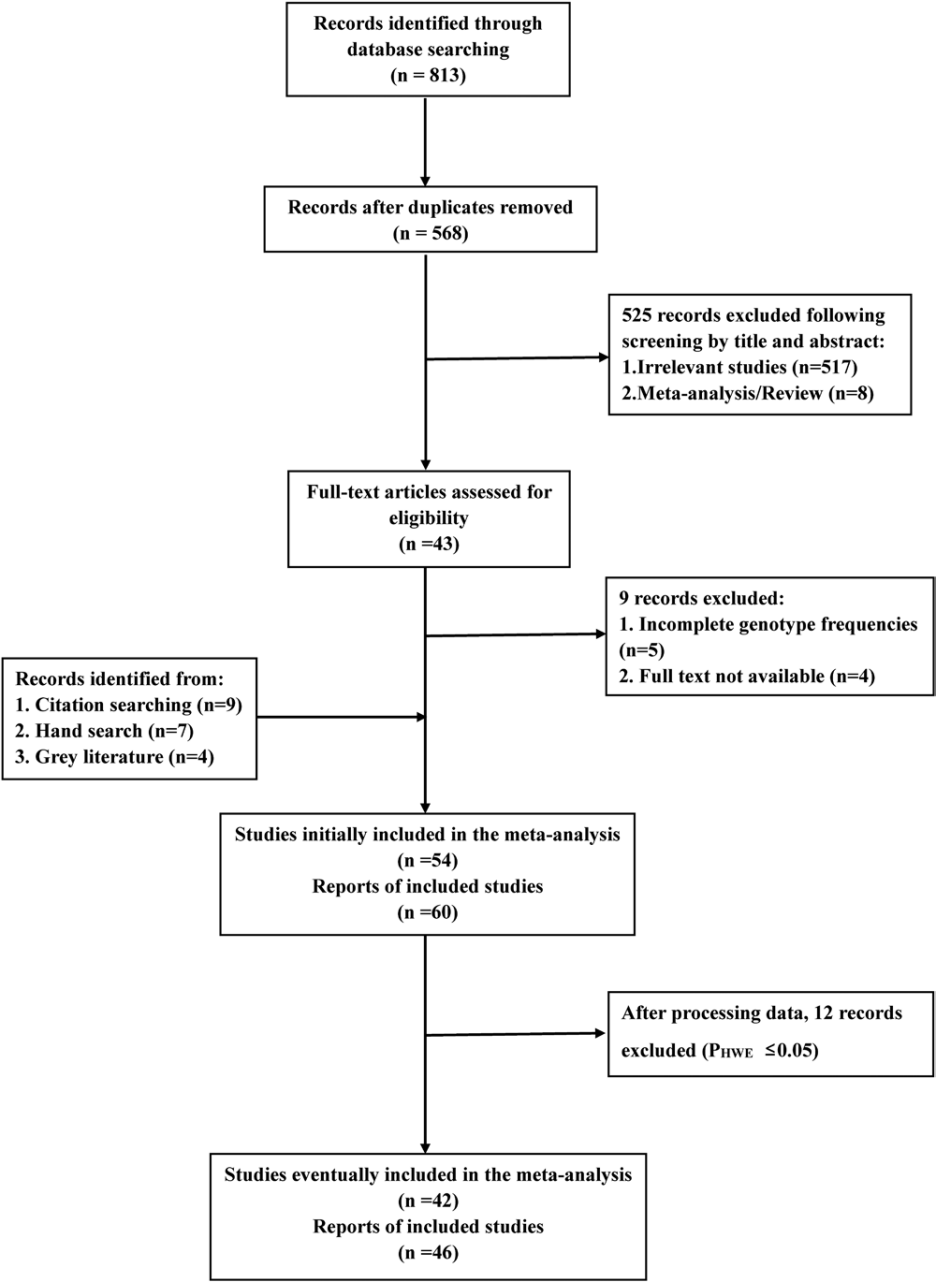
Flow diagram for screening of qualified reports.

54 studies comprising 60 reports were enrolled initially, published literature^[19–68]^ and gray literature.^[69–72]^ We included 46 English-language articles and 8 Chinese-language articles. For the study population, we included 29 studies focused on Asian populations, 20 studies dedicated to Caucasian populations, and 5 studies of other ethnicities (including Omanis, Brazilians, and Tunisians). As for cancer types, there are 4 studies on VEGF-460 and gastric cancer susceptibility, 7 on breast cancer, 4 on prostate cancer, 3 on oral cancer, 3 on colorectal cancer, 2 on colon cancer, 2 on glioma, 3 on cervical cancer, 2 on ovarian cancer, 2 on bladder cancer, 5 on hepatocellular carcinoma, 11 on lung cancer, and 5 on renal cell carcinoma, in addition to studies on endometrial cancer, esophageal adenocarcinoma, pancreatic cancer, nasopharyngeal cancer, osteosarcoma, thyroid cancer, and cutaneous squamous cell carcinoma. We recalculated the HWE of the controls and found 12 studies in which the distribution of control genotype frequencies did not match the HWE (See Table 1).^[19,20,28,32,43,48,49,53,56,58,63,65]^ The deviation from HWE may result from genotyping errors and selection bias in population stratification and control group recruitment.^[98]^ To ensure the accuracy of the results, we excluded these 12 articles. After quality assessment by NOS, 7 studies, including 9 reports, were identified as low quality,^[19,28,32,48,49,58,63]^ the results can be accessed in the Table S2, Supplemental Digital Content, http://links.lww.com/MD/J157 (See Table S2, Supplemental Digital Content, http://links.lww.com/MD/J157, which demonstrates the results of quality evaluation); notably, none of the control gene frequencies of these 7 studies conformed to HWE. All the above information is provided in Table 1. Ultimately, the present meta-analysis enrolled 42 studies comprised 46 reports involving 12868 cases and 14111 controls. The selection process of eligible studies is provided in Figure 1.

**Table 1:** Characteristics of the included studies.

Author and year	Country	Ethnicity	Cancer-type	Control source	Genotype method	Case			Control				
						TT	TC	CC	TT	TC	CC	HWE (controls)	NOS
Chae, 2007	Korea	Asian	Gastric Cancer	PB	PCR-RFLP	218	186	9	225	161	27	0.802	6
Al-Moundhri, 2009	Oman	Omani	Gastric Cancer	Unstated	PCR-RFLP	42	66	22	44	61	25	0.640	7
Jia, 2012	China	Asian	Gastric Cancer	HB	PCR-RFLP	98	56	5	71	78	13	0.178	6
Furuya, 2018	Brazil	Brazilian	Gastric Cancer	PB	Real-time PCR	72	79	27	91	121	41	0.942	7
Kataoka, 2006	Multi-center	Asian	Breast Cancer	PB	TaqMan	616	418	89	665	479	78	0.502	7
Balasubramanian, 2007	England	Caucasian	Breast Cancer	Unstated	TaqMan	111	248	134	106	251	141	0.771	7
Rahoui, 2014	Morocco	Caucasian	Breast Cancer	HB	Real-time PCR	27	25	18	16	39	15	0.337	6
Kapahi, 2015	India	Asian	Breast Cancer	Unstated	PCR-RFLP	61	92	51	72	105	27	0.240	6
Maryam, 2016	Iran	Caucasian	Breast Cancer	PB	PCR-RFLP	69	148	33	66	131	18	**<0.001**	6
Albalawi, 2020	Saudi Arabia	Caucasian	Breast Cancer	PB	ARMS-PCR	54	51	5	31	71	8	**<0.001**	5
Li, 2021	China	Asian	Breast Cancer	Unstated	PCR-LDR	146	98	15	152	103	18	0.922	7
Lin, 2003	China	Asian	Prostate Cancer	HB	PCR-RFLP	60	32	4	43	72	4	**<0.001**	5
Fukuda, 2007	Japan	Asian	Prostate Cancer	HB	PCR-RFLP	143	103	24	132	97	23	0.404	6
Onen, 2007	Turkey	Caucasian	Prostate Cancer	HB	PCR-RFLP	11	89	33	13	94	50	**<0.001**	5
Li, 2017	China	Asian	Prostate Cancer	PB	PCR-RFLP	9	9	10	11	9	10	**0.027**	4
Li, 2017	China	Asian	Bladder Cancer	PB	PCR-RFLP	11	10	9	11	9	10	**0.027**	4
Li, 2017	China	Asian	RCC	PB	PCR-RFLP	16	9	7	11	9	10	**0.027**	4
Ku, 2005	China	Asian	Oral Cancer	HB	PCR-RFLP	120	15	2	38	192	0	**<0.001**	6
Kammerer, 2010	Germany	Caucasian	Oral Cancer	Unstated	Real-time PCR	23	41	16	8	24	8	0.204	7
Borase, 2015	India	Asian	Oral Cancer	Unstated	PCR-RFLP	61	16	3	21	53	6	**<0.001**	5
Maltese, 2009	Italy	Caucasian	Colorectal Cancer	Unstated	PCR-RFLP	70	153	76	47	54	10	0.314	6
Dassoulas, 2009	Greece	Caucasian	Colorectal Cancer	HB	PCR-RFLP	161	104	47	199	121	42	**<0.001**	5
Ehsan, 2021	Iran	Caucasian	Colorectal Cancer	HB	Mass ARRAY	99	116	62	120	178	77	0.462	6
Cacev, 2008	Croatia	Caucasian	Colon Cancer	PB	Real-time PCR	31	84	40	32	83	45	0.574	7
Jannuzzi, 2015	Turkey	Caucasian	Colon Cancer	HB	PCR-RFLP	19	83	1	15	114	0	**<0.001**	5
Linhares, 2018	Portugal	Caucasian	Glioma	HB	Mass ARRAY	3	77	27	41	74	28	0.602	7
Vasconcelos, 2019	Brazil	Brazilian	Glioma	PB	Real-time PCR	83	95	27	72	88	45	0.071	7
Kim, 2010	Korea	Asian	Cervical Cancer	HB	PCR-RFLP	109	83	7	120	77	17	0.359	7
Zidi, 2014	Tunisia	Tunisian	Cervical Cancer	Unstated	Real-time PCR	20	53	13	46	58	20	0.811	6
Konac, 2007	Turkey	Caucasian	Cervical Cancer	HB	PCR-RFLP	15	13	4	13	58	35	0.137	6
Konac, 2007	Turkey	Caucasian	Ovarian Cancer	HB	PCR-RFLP	21	21	5	13	58	35	0.137	6
Konac, 2007	Turkey	Caucasian	Endometrial Cancer	HB	PCR-RFLP	8	8	5	13	58	35	0.137	6
Kazimi, 2010	Turkey	Caucasian	HCC	Unstated	PCR-RFLP	27	28	18	18	24	20	0.075	6
Wu, 2013	China	Asian	HCC	HB	TaqMan	240	146	16	559	406	78	0.719	6
Wu, 2013	China	Asian	HCC	HB	TaqMan	181	135	21	143	132	35	0.589	6
Wu, 2013	China	Asian	HCC	HB	TaqMan	223	126	18	203	136	36	0.069	6
Carvalho, 2021	Brazil	Brazilian	HCC	HB	Real-time PCR	44	59	16	53	54	21	0.260	6
Lee, 2005	Korea	Asian	Lung Cancer	HB	PCR-RFLP	228	184	18	237	168	27	0.700	7
Zhai, 2008	Multi-center	Caucasian	Lung Cancer	HB	TaqMan	539	922	439	422	694	342	0.085	6
Gao, 2012	China	Asian	Lung Cancer	HB	PCR-RFLP	105	80	15	129	68	7	0.583	6
de Mello, 2013	Multi-center	Caucasian	Lung Cancer	HB	MassARRAY	37	79	28	41	72	31	0.954	6
Sun, 2013	China	Asian	Lung Cancer	Unstated	PCR-RFLP	61	43	22	53	69	38	0.100	7
Liu, 2015	China	Asian	Lung Cancer	HB	PCR-RFLP	229	164	21	177	138	23	0.573	7
Yamamoto, 2016	Japan	Asian	Lung Cancer	HB	Real-time PCR	233	197	32	187	157	35	0.825	6
Yu, 2019	China	Asian	Lung Cancer	PB	TaqMan	213	191	29	217	127	36	**0.010**	6
Li, 2014	China	Asian	Lung Cancer	HB	PCR-RFLP	227	159	21	152	103	15	0.649	7
Liu, 2012	China	Asian	Lung Cancer	HB	PCR-RFLP	103	96	61	90	131	39	0.438	7
Yuan, 2011	China	Asian	Lung Cancer	HB	PCR-RFLP	131	101	19	156	90	9	0.351	7
Bruyère, 2010	France	Caucasian	RCC	PB	PCR-RFLP	19	29	1	47	109	46	0.260	7
Sáenz-López, 2013	Spain	Caucasian	RCC	PB	TaqMan	56	111	49	77	138	58	0.793	7
Lu, 2015	China	Asian	RCC	HB	PCR-RFLP	228	93	91	513	168	143	**<0.001**	6
Liu, 2020	China	Asian	RCC	HB	PCR-RFLP	204	168	48	542	258	42	0.128	7
Zhai, 2008	Multi-center	Caucasian	EA	HB	TaqMan	72	155	81	149	257	122	0.582	7
Li, 2010	China	Asian	Ovarian Cancer	HB	PCR-RFLP	198	93	12	191	95	17	0.271	7
Sivaprasad, 2013	India	Asian	Pancreatic Cancer	HB	PCR-RFLP	23	40	17	19	56	12	**0.005**	6
Cheng, 2014	China	Asian	NPC	HB	PCR-RFLP	127	94	19	155	79	11	0.818	7
Zhao, 2015	China	Asian	Osteosarcoma	HB	PCR-RFLP	48	89	39	66	85	25	0.777	6
Bingül, 2016	Turkey	Caucasian	Thyroid Carcinoma	HB	Real-time PCR	49	52	26	75	90	38	0.238	7
Nie, 2016	China	Asian	CSCC	HB	PCR-RFLP	4	31	65	10	63	51	0.111	6
Ai, 2020	China	Asian	Bladder Cancer	HB	Real-time PCR	115	99	16	118	91	21	0.571	7

**Bold text** indicates statistically significant data.

CSCC = cutaneous squamous cell carcinoma, EA = esophageal adenocarcinoma, HB = hospital-based, HCC = hepatocellular carcinoma, HWE = Hardy-Weinberg equilibrium, NOS = Newcastle–Ottawa scale, NPC = nasopharyngeal carcinoma, PB = population-based, RCC = renal cell carcinoma.

### 3.2. Meta-analysis findings

**Figure 2.**
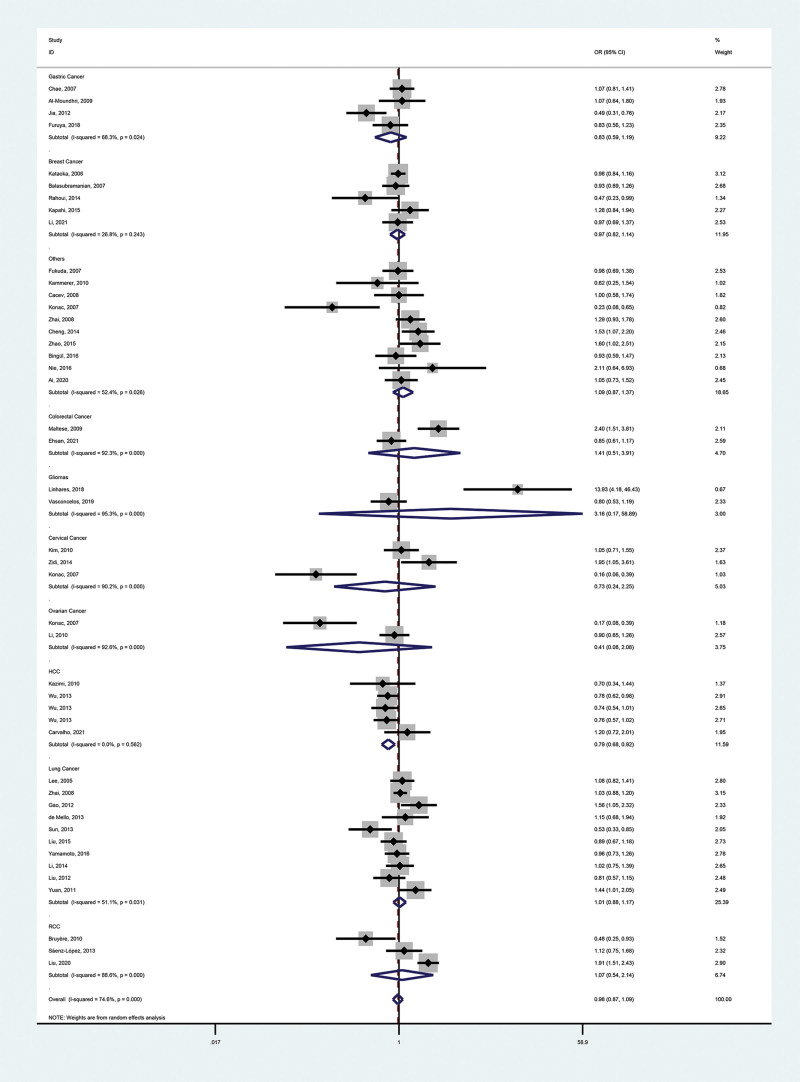
Forest plot of the relationship between VEGF-460(T/C) and cancer risk in the dominant model (TC + CC vs TT). VEGF = vascular endothelial growth factor.

**Figure 3.**
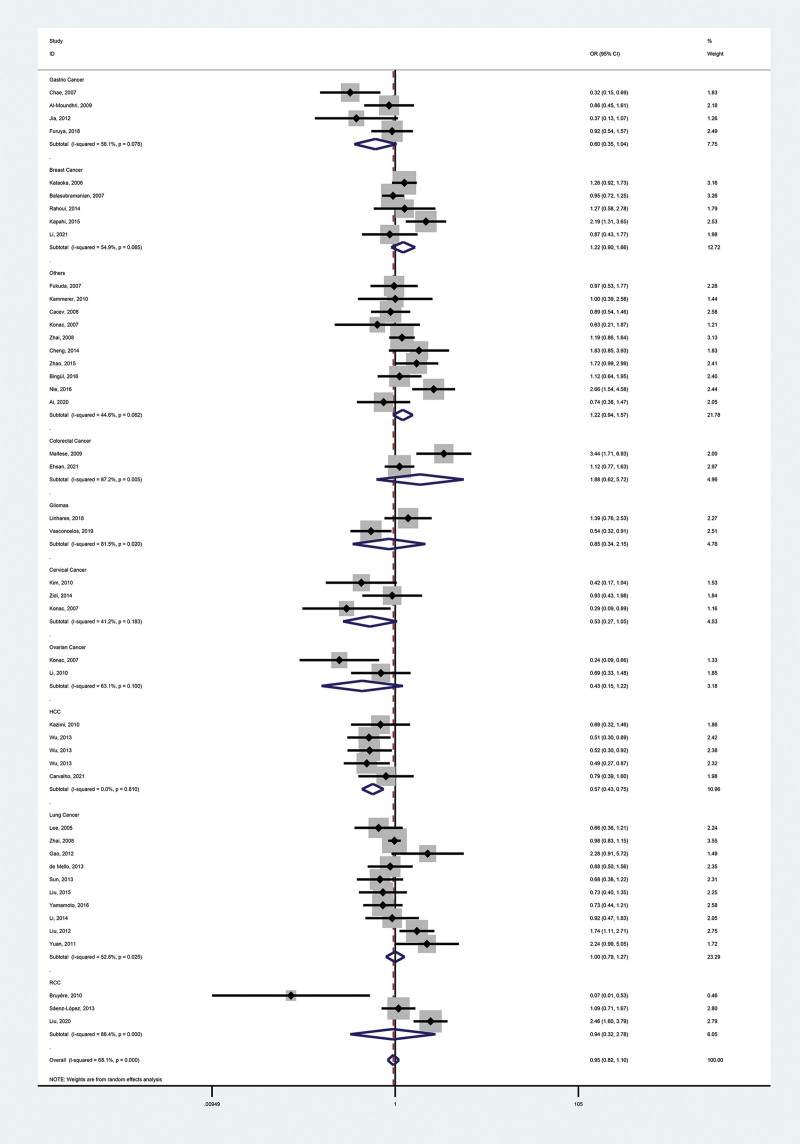
Forest plot of the relationship between VEGF-460(T/C) and cancer risk in the recessive mode (CC vs TC + TT). VEGF = vascular endothelial growth factor.

**Figure 4.**
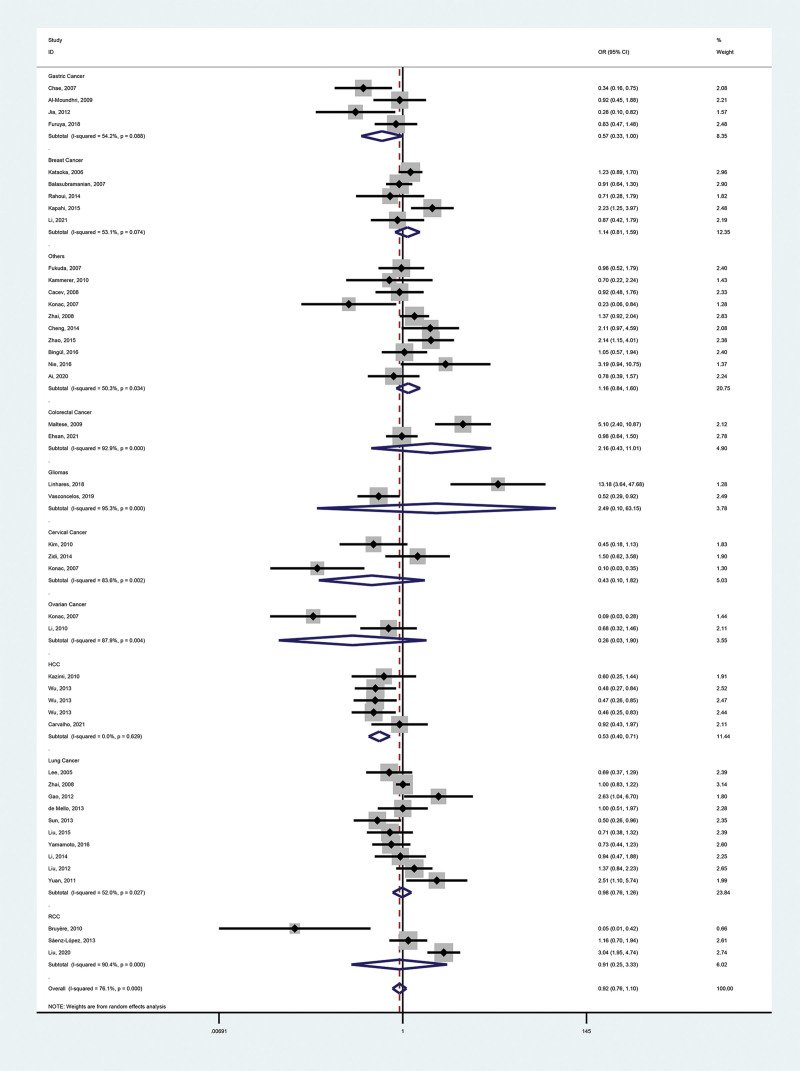
Forest plot of the relationship between VEGF-460(T/C) and cancer risk in the homozygous model (CC vs TT). VEGF = vascular endothelial growth factor.

**Figure 5.**
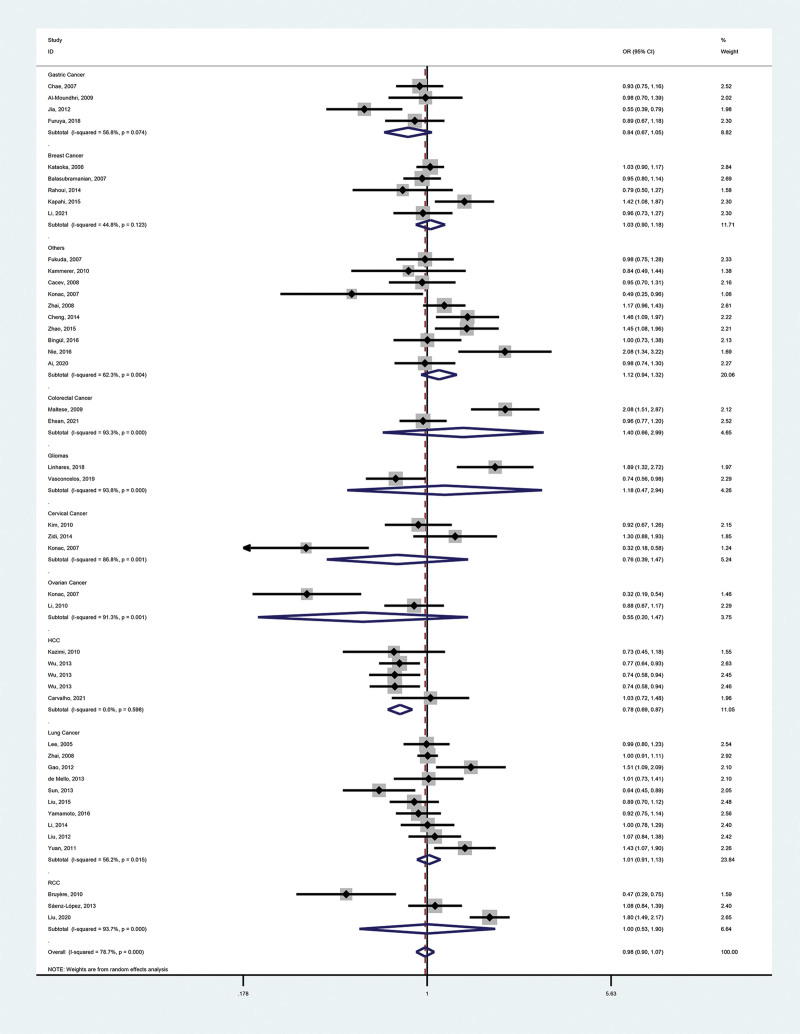
Forest plot of the relationship between VEGF-460(T/C) and cancer risk in the additive model (C vs T). VEGF = vascular endothelial growth factor.

These meta-analysis findings are provided in Tables 2, 3, and Figures 2–5.

**Table 2:** Association of VEGF-460 (T/C) and cancer risk in the cancer type subgroup.

Cancer type	N	Dominant model (TC + CC vs TT)		Recessive model (CC vs TC + TT)		Heterozygous model (TC vs TT)		Homozygous model (CC vs TT)		Additive model (C vs T)	
		OR (95% CI)	*P*/*I*^2^ (%)	OR (95% CI)	*P*/*I*^2^ (%)	OR (95% CI)	*P*/*I*^2^ (%)	OR (95% CI)	*P*/*I*^2^ (%)	OR (95% CI)	*P*/*I*^2^ (%)
Overall	46	0.98 (0.87, 1.09)	<.001/74.6%	0.95 (0.82, 1.10)	<.001/68.1%	0.99 (0.90, 1.10)	<.001/65.9%	0.92 (0.76, 1.10)	<.001/76.1%	0.98 (0.90, 1.07)	<.001/78.7%
Gastric Cancer	4	0.83 (0.59, 1.19)	.024/68.3%	0.60 (0.35, 1.04)	.078/56.1%	0.88 (0.61, 1.29)	.019/69.8%	0.57 (0.33, 1.00)	.088/54.2%	0.84 (0.67, 1.05)	.074/56.8%
Breast Cancer	5	0.97 (0.82, 1.14)	.243/26.8%	1.22 (0.90, 1.66)	.065/54.9%	0.93 (0.78, 1.10)	.265/23.4%	1.14 (0.81, 1.59)	.074/53.1%	1.03 (0.90, 1.18)	.123/44.8%
Colorectal Cancer	2	1.41 (0.51, 3.91)	<.001/92.3%	1.88 (0.62, 5.72)	.005/87.2%	1.21 (0.51, 2.85)	.004/87.9%	2.16 (0.43, 11.01)	<.001/92.9%	1.40 (0.66, 2.99)	<.001/93.3%
Gliomas	2	3.16 (0.17, 58.89)	<.001/95.3%	0.85 (0.34, 2.15)	.020/81.5%	3.45 (0.22, 54.60)	<.001/94.6%	2.49 (0.10, 63.15)	<.001/95.3%	1.18 (0.47, 2.94)	<.001/93.8%
Cervical Cancer	3	0.73 (0.24, 2.25)	<.001/90.2%	0.53 (0.27, 1.05)	.183/41.2%	0.84 (0.29, 2.45)	<.001/88.0%	0.43 (0.10, 1.82)	.002/83.6%	0.76 (0.39, 1.47)	.001/86.8%
Ovarian Cancer	2	0.41 (0.08, 2.08)	<.001/92.6%	0.43 (0.15, 1.22)	.100/63.1%	0.49 (0.12, 1.98)	.002/89.3%	0.26 (0.03, 1.90)	.004/87.9%	0.55 (0.20, 1.47)	.001/91.3%
HCC	5	**0.79 (0.68, 0.92**)	.562/0.0%	**0.57 (0.43, 0.75**)	.810/0.0%	0.86 (0.74, 1.00)	.625/0.0%	**0.53 (0.40, 0.71**)	.629/0.0%	**0.78 (0.69, 0.87**)	.598/0.0%
Lung Cancer	10	1.01 (0.88, 1.17)	.031/51.1%	1.00 (0.79, 1.27)	.025/52.8%	1.01 (0.88, 1.17)	.043/48.3%	0.98 (0.76, 1.26)	.027/52.0%	1.01 (0.91, 1.13)	.015/56.2%
RCC	3	1.07 (0.54, 2.14)	<.001/88.6%	0.94 (0.32, 2.78)	<.001/88.4%	1.16 (0.70, 1.94)	.013/77.0%	0.91 (0.25, 3.33)	<.001/90.4%	1.00 (0.53, 1.90)	<.001/93.7%
Others	10	1.09 (0.87, 1.37)	.026/52.4%	1.22 (0.94, 1.57)	.062/44.6%	1.08 (0.88, 1.32)	.125/35.4%	1.16 (0.84, 1.60)	.034/50.3%	1.12 (0.94, 1.32)	.004/62.3%

**Bold text** indicates statistically significant data.

HCC = hepatocellular carcinoma, Others = prostate cancer, oral cancer, colon cancer, endometrial cancer, esophageal adenocarcinoma, nasopharyngeal carcinoma, osteosarcoma, thyroid carcinoma, cutaneous squamous cell carcinoma, and bladder cancer, RCC = renal cell carcinoma, VEGF = vascular endothelial growth factor.

**Table 3:** Association between VEGF-460 (T/C) and cancer risk in other subgroups.

Subgroups	N	Dominant model (TC + CC vs TT)		Recessive model (CC vs TC + TT)		Heterozygous model (TC vs TT)		Homozygous model (CC vs TT)		Additive model (C vs T)	
		OR (95% CI)	*P*/*I*^2^ (%)	OR (95% CI)	*P*/*I*^2^ (%)	OR (95% CI)	*P*/*I*^2^ (%)	OR (95% CI)	*P*/*I*^2^ (%)	OR (95% CI)	*P*/*I*^2^ (%)
Overall	46	0.98 (0.87, 1.09)	<.001/74.6%	0.95 (0.82, 1.10)	<.001/68.1%	0.99 (0.90, 1.10)	<.001/65.9%	0.92 (0.76, 1.10)	<.001/76.1%	0.98 (0.90, 1.07)	<.001/78.7%
Ethnicity											
Asian	24	1.02 (0.90, 1.15)	<.001/71.6%	0.98 (0.76, 1.26)	<.001/76.4%	1.02 (0.91, 1.13)	<.001/59.3%	0.96 (0.73, 1.26)	<.001/77.3%	1.03 (0.92, 1.15)	<.001/80.5%
Caucasian	17	0.84 (0.64, 1.09)	<.001/81.9%	0.98 (0.81, 1.19)	.003/56.1%	0.86 (0.67, 1.11)	<.001/76.5%	0.86 (0.62, 1.20)	<.001/80.2%	0.90 (0.77, 1.06)	<.001/81.4%
Others	5	1.04 (0.78, 1.39)	.127/44.2%	0.77 (0.59, 1.01)	.630/0.0%	1.13 (0.84, 1.51)	.156/39.9%	0.82 (0.59, 1.12)	.352/9.6%	0.94 (0.76, 1.12)	.220/30.2%
Control source											
PB	7	0.95 (0.83, 1.08)	.327/13.5%	0.74 (0.50, 1.11)	.001/73.3%	0.98 (0.87, 1.10)	.614/0.0%	0.72 (0.47, 1.11)	.001/72.2%	0.90 (0.77, 1.04)	.026/58.2%
HB	30	0.97 (0.84, 1.12)	<.001/79.4%	0.96 (0.80, 1.16)	<.001/69.1%	0.98 (0.85, 1.12)	<.001/73.2%	0.91 (0.72, 1.17)	<.001/78.3%	0.99 (0.88, 1.11)	<.001/81.6%
Unstated	9	1.06 (0.78, 1.43)	<.001/71.6%	1.10 (0.78, 1.56)	.003/66.1%	1.04 (0.80, 1.35)	.016/57.4%	1.13 (0.71, 1.78)	<.001/75.0%	1.05 (0.84, 1.32)	<.001/77.7%
Genotype method											
PCR-RFLP	24	0.94 (0.77, 1.15)	<.001/81.9%	0.97 (0.72, 1.32)	<.001/78.1%	0.96 (0.81, 1.14)	<.001/73.6%	0.87 (0.59, 1.27)	<.001/83.3%	0.98 (0.83, 1.15)	<.001/85.8%
Real-time PCR	10	0.95 (0.80, 1.12)	.221/24.2%	0.84 (0.69, 1.02)	.774/0.0%	0.98 (0.81, 1.20)	.109/37.4%	0.81 (0.65, 1.00)	.798/0.0%	0.93 (0.84, 1.03)	.682/0.0%
TaqMan	8	0.93 (0.83, 1.05)	.077/45.3%	0.89 (0.72, 1.10)	.006/64.3%	0.96 (0.88, 1.05)	.467/0.0%	0.86 (0.67, 1.12)	.001/70.7%	0.93 (0.84, 1.04)	.004/66.8%
PCR-LDR	1	0.97 (0.69, 1.37)	–/–	0.87 (0.43, 1.77)	–/–	0.99 (0.69, 1.42)	–/–	0.87 (0.42, 1.79)	–/–	0.96 (0.73, 1.27)	–/–
Mass ARRAY	3	1.97 (0.67, 5.77)	<.001/90.3%	1.11 (0.84, 1.46)	.561/0.0%	2.01 (0.64, 6.34)	<.001/90.7%	1.97 (0.63, 6.14)	.001/86.5%	1.21 (0.81, 1.79)	.006/80.6%
Literature type											
Published literature	42	0.97 (0.86, 1.09)	<.001/76.1%	0.93 (0.80, 1.08)	<.001/68.7%	0.99 (0.89, 1.10)	<.001/67.0%	0.89 (0.73, 1.08)	<.001/77.4%	0.97 (0.88, 1.06)	<.001/80.0%
Grey literature	4	1.06 (0.84, 1.33)	.155/42.7%	1.28 (0.79, 2.08)	.076/56.3%	1.00 (0.75, 1.34)	.049/61.8%	1.23 (0.79, 1.91)	.154/42.9%	1.10 (0.94, 1.29)	.223/29.9%

Others = Omani, Brazilian, and Tunisian, VEGF = vascular endothelial growth factor.

#### 3.2.1. Primary outcome.

We identified no association between polymorphisms and malignancy risk (dominant model, OR = 0.98, 95% CI = 0.87–1.09; recessive model, OR = 0.95, 95% CI = 0.82–1.10; heterozygous model, OR = 0.99, 95% CI = 0.90–1.10; homozygous model, OR = 0.92, 95% CI = 0.76–1.10; additive model, OR = 0.98, 95% CI = 0.90–1.07).

#### 3.2.2. Secondary outcomes.

To further investigate the correlation between VEGF-460 polymorphism and cancers, we performed stratification based on cancer type, ethnicity, control sources, genotyping methods, and literature type. In the cancer type subgroup, we found that this SNP reduced the risk of hepatocellular carcinoma (HCC; dominant model, OR = 0.79, 95% CI = 0.68–0.92; recessive model, OR = 0.57, 95% CI = 0.43–0.75; homozygous model, OR = 0.53, 95% CI = 0.40–0.71; additive model, OR = 0.78, 95% CI = 0.69–0.87). Notably, the HCC subgroup contained 5 reports, 3 of which had controls who were chronic hepatitis B (CHB) patients (a risk factor for HCC), yet concluded that the SNP was a protective factor for HCC, which strengthens the credibility of the conclusion. VEGF-460 polymorphism was not associated with other types of malignancies. In all other subgroups, the SNP was not associated with cancer.

Overall, the SNP is not relevant to overall cancer but may lower the risk of HCC.

### 3.3. Heterogeneity analysis

High heterogeneity was present in 5 models (Tables 2 and 3 provide P_Heterogeneity_ and *I*^2^). We performed meta-regression based on cancer type, ethnicity, the sources of controls, genotyping methods, and literature publication status, and the results were not statistically significant (Table 4). Therefore, we further explored the origins of heterogeneity using subgroup analysis. All showed heterogeneity in the cancer type subgroup except for the breast cancer and HCC groups. Among ethnicity, sources of controls, genotyping methods, and literature type subgroups, heterogeneity was detected in all subgroups except the real-time PCR and gray literature groups.

**Table 4:** Results of meta-regression analysis of VEGF-460 (T/C) and cancer risk in 5 genetic models.

Covariates	Number of dummy variables	Dominant model	Recessive model	Heterozygous model	Homozygous model	Additive model
Year	–	0.327	0.190	0422	0.195	0.217
Ethnicity	3	0.279	0.709	0.321	0.749	0.391
Cancer type	10	0.898	0.279	0.765	0.478	0.450
Genotyping methods	5	0.342	0.619	0.394	0.538	0.695
Source of controls	3	0.842	0.500	0.682	0.732	0.770
Literature type	2	0.872	0.358	0.926	0.498	0.667

VEGF = vascular endothelial growth factor.

Overall, this work had high heterogeneity due to multifactorial factors, including differences in cancer type, the population, source of controls, genotyping methods, and literature type in the included studies.

### 3.4. Publication bias

**Figure 6.**
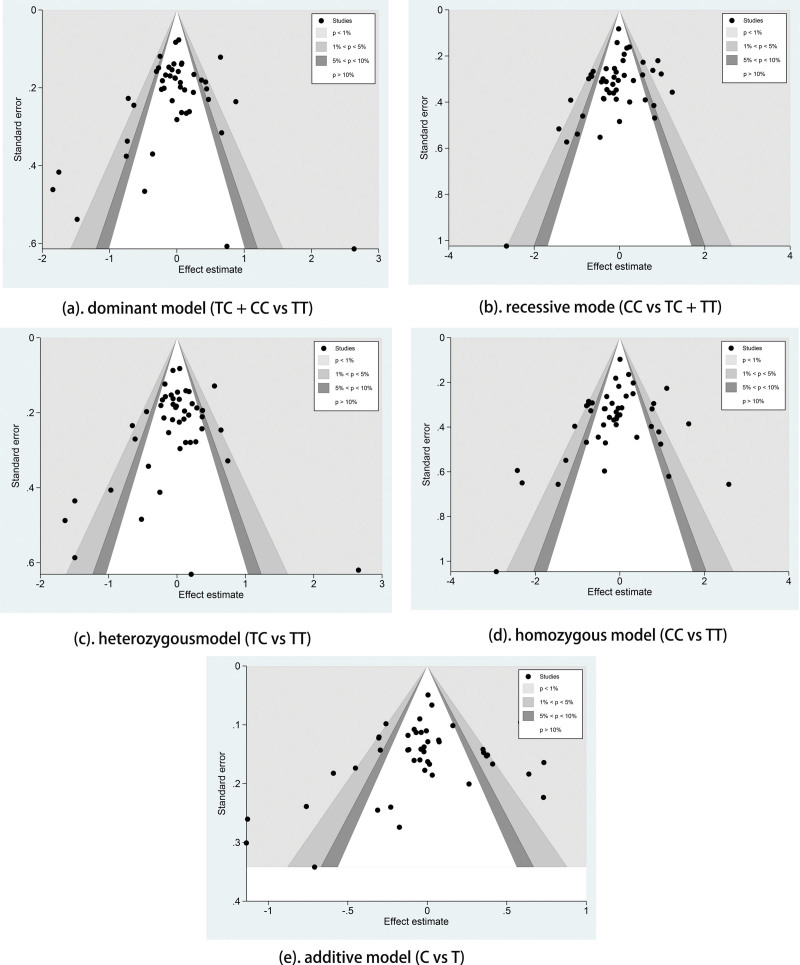
Funnel plot of the relationship between VEGF-460 and cancer risk. (A) dominant model, (B) recessive model, (C) heterozygous model, (D) homozygous model, (E) additive model. VEGF = vascular endothelial growth factor.

We used contour-enhanced funnel plots to estimate the publication bias in 5 gene models and found that all 5 funnel plots were approximately symmetrical (Fig. 6). The funnel plots had studies distributed in statistically significant places outside the white areas, indicating study heterogeneity, consistent with P_Heterogeneity_ and *I*^2^ value. And the results of Begg’s and Egger’s tests showed the presence of publication bias in the recessive model (dominant model: P_Begg_ = 0.379, P_Egger_ = 0.512; recessive model: P_Begg_ < 0.001, P_Egger_ < 0.001; heterozygous model: P_Begg_ = 0.622, P_Egger_ = 0.077; homozygous model: P_Begg_ = 0.214, P_Egger_ = 0.548; additive model: P_Begg_ = 0.520, P_Egger_ = 0.967). The effects of publication bias were assessed by the trim and fill method for the recessive model, and we found no significant alteration in the results, suggesting that the results were steady and the publication bias was acceptable.

### 3.5. Sensitivity analysis

After we rotated out individual studies to recombine ORs, we found no significant alteration in results, much less reversal. The sensitivity analysis results on the 5 models are provided in Figure S1, Supplemental Digital Content, http://links.lww.com/MD/J158 (See Figure S1, Supplemental Digital Content, http://links.lww.com/MD/J158, which demonstrates the results of sensitivity analysis in 5 model). The above results indicated the stability of the results of this paper.

## 4. Discussion

The present meta-analysis included 44 articles comprising 46 case-control trials with 12,868 cases and 14,111 controls. We found that VEGF-460(T/C) polymorphism was unrelated to overall malignancy but may reduce the risk of HCC. Four previous meta-analyses were performed on this SNP and lung cancer risk; Junwei Tu et al, Fengming Yang et al and Junli Fan et al concluded that this SNP increases the lung cancer risk among Asians^[81–83]^; nevertheless, Ning Song et al identified VEGF-460(T/C) polymorphism as a defensive factor in nonsmokers and patients with squamous lung cancer.^[80]^ Zhou et al^[84]^ concluded that the SNP was not relevant to colorectal cancer, whereas Zigang Zhao et al concluded that it could result in colorectal cancer.^[85]^ Both meta-findings demonstrate that this SNP is unrelated to renal cell carcinoma.^[87,88]^ And J. Zhao et al conclude that VEGF-460 enhances the risk of osteosarcoma.^[91]^ As for ovarian, gastric, oral, and prostate cancers, there is no evidence of VEGF-460 being related to them.^[86,89,90,92]^ A meta-analysis of VEGF-460 and overall cancer susceptibility was reported in 2009 by Xu et al.^[79]^ They concluded that this SNP could contribute to elevated cancer risk in Asian populations. Most of the above meta-analysis conclusions were consistent with ours, but only for single cancer and less included literature. This meta-analysis included 30 more eligible papers than the previous meta-analysis examining VEGF-460 and overall cancer risk.

This meta-analysis incorporated a large body of case-control studies targeting different malignancies, ethnic populations, recruitment methods, etc. Therefore, heterogeneity detection is inevitable. In order to minimize the effect of heterogeneity, random-effects models were taken once the heterogeneity was detected. Meta-regression based on year (year of publication or graduation), ethnicity, cancer type, genotyping method, control sources, and literature type was performed to probe sources of heterogeneity. However, *P* values for all covariates exceeded .05, indicating that no sources of heterogeneity were detected. We then performed a detailed subgroup analysis and found heterogeneity in almost all subgroups, suggesting multiple causes of heterogeneity, including differences in tumor type, race, participant recruitment methods, and genotyping methods.

We included gray literature to reduce publication bias as much as possible. We initially used contour-enhanced funnel plots to detect publication bias, roughly symmetrical for the 5 models. However, a few studies were distributed in statistically significant regions outside the white areas, which is not the result of publication bias but most likely due to heterogeneity. The Begg’s and Egger’s tests were employed to quantify publication bias, and the results indicated that publication bias was mainly present in the recessive model. We performed the trim and fill method for the recessive model and found no change in the results after trim and fill, indicating that the publication bias was acceptable. The sensitivity analysis was conducted by eliminating single reports in turn, then recombining the ORs to test the stability of the results. The ORs and 95% CIs showed no significant changes and no reversal, indicating that these meta-analysis findings were steady.

Since Judah Folkman first emphasized the involvement of angiogenesis in the growth and multiplication of solid tumors in 1971,^[99]^ the concept of anti-angiogenesis as a potential means of cancer therapy has been repeatedly proposed.^[100,101]^ With the follow-up of related studies, anti-angiogenic drugs, especially those targeting VEGF, like bevacizumab, sunitinib, and pazopanib, have been used for oncology therapy.^[102]^ Despite the effectiveness of these clinically approved anti-angiogenic drugs in decreasing cancer angiogenesis through normalization of hyperpermeable tumor vessels, metastasis and death still follow therapy.^[102]^ This is due to the unclear mechanism of VEGF involvement in oncogenesis and proliferation and the resistance to anti-angiogenic drugs. Future research may be multi-directional, such as anti-angiogenic drugs combined with immunotherapy or nanoparticles.^[103–105]^ However, research on VEGF mechanisms and other pro-angiogenic factors involved in tumor formation and proliferation must be fundamental and essential.

This Meta-analysis incorporated more studies with different types of cancer, including unpublished literature, compared to previous meta-analyses of the same type, and the results were more comprehensive and reliable. Although it was concluded that VEGF-460 did not correlate with malignancy risk, the SNP might reduce HCC susceptibility in the subgroup of cancer types. It is noteworthy that a large proportion of controls in this subgroup were patients with chronic hepatitis B, one of the factors contributing to HCC; if the result is this SNP causing HCC, then the phenomenon reduces the validity of the result, and vice versa increases the validity of the opposite result. This may be a breakthrough for related research in the future.

However, some limitations remain; first, there was high heterogeneity due to multiple factors. Secondly, the African population was not analyzed. Third, because of the limitations of the original study, it was not possible to analyze on more detailed stratification, such as gender, BMI, whether or not to smoke.

In conclusion, VEGF-460(T/C) was not associated with malignancy. However, in the cancer type subgroup, this SNP reduced the risk of HCC. Future studies with more rigorous trial designs and larger sample sizes are required to update and refine our conclusions.

## 5. Conclusion

This meta-analysis indicated that VEGF-460 was irrelevant to overall malignancy risk, but it might be a protective factor for HCC.

This work was funded by the Youth Science Fund Project of Jiangxi Provincial Department of Science and Technology (20192BAB215037).

## Author contributions

**Conceptualization:** Foyan Xu.

**Data curation:** Haoran Qin, Taiping Li.

**Formal analysis:** Haoran Qin, Qiang Xiao, Yufen Xie, Dan Li.

**Funding acquisition:** Foyan Xu.

**Investigation:** Haoran Qin, Qiang Xiao, Yufen Xie, Xiaozhou Long, Yiqin Liu.

**Methodology:** Haoran Qin, Dan Li, Xiaozhou Long, Taiping Li.

**Supervision:** Foyan Xu.

**Validation:** Qiang Xiao, Yufen Xie, Siqing Yi, Jian Chen.

**Writing – original draft:** Haoran Qin, Qiang Xiao.

**Writing – review & editing:** Jian Chen.

## Supplementary Material
