# Supplementary material for: The relationship between VEGF-460(T>C) polymorphism and cancer risk: A systematic review and meta-analysis based on 46 reports
Source: Medicine (Baltimore). 2023 Jun 30;102(26):e34089. doi: 10.1097/MD.0000000000034089 (PMC10313293; doi:10.1097/MD.0000000000034089)
Supplement: Supplementary file 1 [file medi-102-e34089-s001.pdf]

**Supplementary Table 1**

PubMed Search Strategy

| |
|---|
| (((((((((((((("Neoplasms"[Mesh]) OR (Neoplasia[Title/Abstract])) OR (Neoplasias[Title/Abstract])) OR (Neoplasm[Title/Abstract])) OR (Tumors[Title/Abstract])) OR (Tumor[Title/Abstract])) OR (Cancer[Title/Abstract])) OR (Cancers[Title/Abstract])) OR (Malignancy[Title/Abstract])) OR (Malignancies[Title/Abstract])) OR (Malignant Neoplasms[Title/Abstract])) OR (Malignant Neoplasm[Title/Abstract])) OR (Neoplasm, Malignant[Title/Abstract])) OR (Neoplasms, Malignant[Title/Abstract]) |
| AND |
| ((("Vascular Endothelial Growth Factors"[Mesh]) OR (VEGFs[Title/Abstract])) OR (VEGF-460[Title/Abstract])) OR (rs833061[Title/Abstract]) |
| AND |
| ((((((((((("polymorphism, single nucleotide"[MeSH]) OR ("Mutation"[Mesh])) OR ("Genetic Variation"[Mesh])) OR ("Alleles"[Mesh])) OR (nucleotide polymorphism single[Title/Abstract])) OR (nucleotide polymorphisms single[Title/Abstract])) OR (polymorphisms single nucleotide[Title/Abstract])) OR (single nucleotide polymorphisms[Title/Abstract])) OR (SNPs[Title/Abstract])) OR (single nucleotide polymorphism[Title/Abstract])) OR (Polymorphism[Title/Abstract]) |
| AND |
| ((("Case-Control Studies"[Mesh]) OR (Case-Control Study[Publication Type])) OR (Studies, Case-Control[Publication Type])) OR (Study, Case-Control[Publication Type]) |

**Supplementary Table S2.** Embase Search Strategy

| |
|---|
| 'neoplasm'/exp OR neoplasms:ti,ab,kw OR neoplasia:ti,ab,kw OR neoplasias:ti,ab,kw OR tumors:ti,ab,kw OR tumor:ti,ab,kw OR cancer:ti,ab,kw OR cancers:ti,ab,kw OR malignancy:ti,ab,kw OR malignancies:ti,ab,kw OR 'malignant neoplasms':ti,ab,kw OR 'malignant neoplasm':ti,ab,kw OR 'neoplasm, malignant':ti,ab,kw OR 'neoplasms, malignant':ti,ab,kw |
| AND |
| 'vascular endothelial growth factors':ti,ab,kw OR vegfs:ti,ab,kw OR rs833061:ti,ab,kw OR 'vegf 460':ti,ab,kw |
| AND |
| 'single nucleotide polymorphism'/exp OR 'nucleotide polymorphism, single':ti,ab,kw OR 'nucleotide polymorphisms, single':ti,ab,kw OR 'polymorphism, single nucleotide':ti,ab,kw OR 'polymorphisms, single nucleotide':ti,ab,kw OR 'single nucleotide polymorphisms':ti,ab,kw OR snps:ti,ab,kw OR polymorphism:ti,ab,kw OR allele:ti,ab,kw OR variation:ti,ab,kw |
| AND |
| 'case control study'/exp OR 'case-control studies':ti,ab,kw OR 'case-control study':ti,ab,kw OR 'case control':ti,ab,kw |

**Supplementary Table S3.** Web of Science Search Strategy

| |
|---|
| TS=(Neoplasms OR Neoplasia OR Neoplasias OR Neoplasm OR Tumors OR Tumor OR Cancer OR Cancers OR Malignancy OR Malignancies OR Malignant Neoplasms OR Malignant Neoplasm OR Neoplasm, Malignant OR Neoplasms, Malignant) |
| AND |
| TS=(Vascular Endothelial Growth Factors OR VEGFs OR rs833061 OR VEGF-460) |
| AND |
| TS=(Polymorphism, Single Nucleotide OR Nucleotide Polymorphism, Single OR Nucleotide Polymorphisms, Single OR Polymorphisms, Single Nucleotide OR Single Nucleotide Polymorphisms OR SNPs OR Single Nucleotide Polymorphism OR Polymorphism OR Allele OR Variation) |
| AND |
| TS=(Case-Control Studies OR Case-Control Study OR Studies, Case-Control OR Study, Case-Control) |

**Supplementary Table S4**. CNKI Search Strategy

（主题：肿瘤）OR（主题：癌症）OR（主题：恶性肿瘤）OR（主题：癌）

AND

（主题：血管内皮生长因子）OR（主题：VEGF-460）OR（主题：rs833061）

AND

（主题：单核苷酸多态性）OR（主题：多态性）

AND

（摘要：病例对照研究）OR（摘要：基于病例研究）OR（摘要：病例比较研究）OR（摘要：病例关联研究） OR（摘要：配对病例对照研究）OR（摘要：成批病例对照研究）

**English version:**

**Supplementary Table S4**. CNKI Search Strategy

(Theme: Tumor) OR (Theme: Cancer) OR (Theme: Malignancy) OR (Theme: Carcinoma)

AND

(Theme: Vascular Endothelial Growth Factor) OR (Theme: VEGF-460) OR (Theme: rs833061)

AND

(Theme: Single Nucleotide Polymorphism) OR (Theme: Polymorphism)

AND

(Abstract: case-control study) OR (Abstract: case-based study) OR (Abstract: case-comparison study) OR (Abstract: case-association study) OR (Abstract: paired case-control study) OR (Abstract: batch case-control study)

**Supplementary Table S5.** Wangfang database Search Strategy

| |
|---|
| 主题:("肿瘤") or 主题:("癌症") or 主题:("恶性肿瘤") or 主题:("癌") |
| AND |
| 主题:("血管内皮生长因子") or 主题:("VEGF-460") or 主题:("rs833061") |
| AND |
| 主题:("单核苷酸多态性") or 主题:("基因多态性") |
| AND |
| 摘要:("病例对照研究") or 摘要:("基于病例研究") or 摘要:("病例比较研究") or 摘要:("病例关联研究") or 摘要:("配对病例对照研究") or 摘要:("成批病例对照研究") |

**English version:**

**Supplementary Table S5.** Wangfang database Search Strategy

| |
|---|
| Theme:("Tumor") or Theme:("Cancer") or Theme:("Malignancy") or Theme:(" Carcinoma") |
| AND |
| Theme:("Vascular Endothelial Growth Factor") or Theme:("VEGF-460") or Theme:("rs833061") |
| AND |
| Theme:("Single Nucleotide Polymorphism") or Theme:("Gene Polymorphism") |
| AND |
| Abstract: ("Case-Control Study") or Abstract: ("Case-Based Study") or Abstract: ("Case-Comparison Study") or Abstract: ("Case-Association Study") or Abstract: ("Paired Case-Control Study") or Abstract: ("Batch Case-Control Study") |

**Supplementary Table S6.** Quality assessment of the included case-control studies by the Newcastle-Ottawa scale (NOS)

| Author and Year | Selection | | | | Comparability | Exposure | | | Score |
|---|---|---|---|---|---|---|---|---|---|
| | Criteria 1 | Criteria 2 | Criteria 3 | Criteria 4 | Criteria 5 | Criteria 6 | Criteria 7 | Criteria 8 | |
| Chae, 2007 | ★ | ★ | ★ | ★ | ★ | - | ★ | - | 6 |
| Al-Moundhri, 2009 | ★ | ★ | - | ★ | ★★ | ★ | ★ | - | 7 |
| Jia, 2012 | ★ | ★ | - | ★ | ★★ | - | ★ | - | 6 |
| Furuya, 2018 | ★ | ★ | ★ | ★ | ★★ | - | ★ | - | 7 |
| Kataoka, 2006 | ★ | ★ | ★ | ★ | ★★ | - | ★ | | 7 |
| Balasubramanian, 2007 | ★ | ★ | - | ★ | ★★ | ★ | ★ | - | 7 |
| Rahoui, 2014 | ★ | ★ | - | ★ | ★★ | - | ★ | - | 6 |
| Kapahi, 2015 | ★ | ★ | - | ★ | ★★ | - | ★ | - | 6 |
| Maryam, 2016 | ★ | ★ | ★ | ★ | ★ | - | ★ | - | 6 |
| Albalawi, 2020 | ★ | - | ★ | ★ | ★ | - | ★ | - | 5 |
| Li, 2021 | ★ | ★ | - | ★ | ★★ | ★ | ★ | - | 7 |
| Lin, 2003 | ★ | - | - | ★ | ★★ | - | ★ | - | 5 |
| Fukuda, 2007 | ★ | ★ | - | ★ | ★★ | - | ★ | - | 6 |
| Onen, 2007 | ★ | - | - | ★ | ★★ | - | ★ | - | 5 |
| Li, 2017 | ★ | - | - | ★ | ★ | - | ★ | - | 4 |
| Ku, 2005 | ★ | ★ | - | ★ | ★ | ★ | ★ | - | 6 |
| Kammerer, 2010 | ★ | ★ | - | ★ | ★★ | ★ | ★ | - | 7 |
| Borase, 2015 | ★ | - | - | ★ | ★★ | - | ★ | - | 5 |
| Maltese, 2009 | ★ | ★ | - | ★ | ★★ | - | ★ | - | 6 |
| Dassoulas, 2009 | ★ | - | - | ★ | ★★ | - | ★ | - | 5 |
| Ehsan, 2021 | ★ | ★ | - | ★ | ★★ | - | ★ | - | 6 |
| Cacev, 2008 | ★ | ★ | ★ | ★ | ★★ | - | ★ | - | 7 |
| Jannuzzi, 2015 | ★ | - | - | ★ | ★★ | - | ★ | - | 5 |
| Linhares, 2018 | ★ | ★ | - | ★ | ★★ | ★ | ★ | - | 7 |
| Vasconcelos, 2019 | ★ | ★ | ★ | ★ | ★ | ★ | ★ | - | 7 |
| Kim, 2010 | ★ | ★ | - | ★ | ★★ | ★ | ★ | - | 7 |
| Zidi, 2014 | ★ | ★ | - | ★ | ★★ | - | ★ | - | 6 |
| Konac, 2007 | ★ | ★ | - | ★ | ★★ | - | ★ | - | 6 |
| Kazimi, 2010 | ★ | ★ | - | ★ | ★★ | - | ★ | - | 6 |
| Wu, 2013 | ★ | ★ | - | - | ★★ | ★ | ★ | - | 6 |
| Carvalho, 2021 | ★ | ★ | - | ★ | ★★ | - | ★ | - | 6 |
| Lee, 2005 | ★ | ★ | - | ★ | ★★ | ★ | ★ | - | 7 |
| Zhai, 2008 | ★ | ★ | - | ★ | ★★ | - | ★ | - | 6 |
| Gao, 2012 | ★ | ★ | - | ★ | ★★ | - | ★ | - | 6 |
| de Mello, 2013 | ★ | ★ | - | ★ | ★★ | - | ★ | - | 6 |
| Sun, 2013 | ★ | ★ | - | ★ | ★★ | ★ | ★ | - | 7 |
| Liu, 2015 | ★ | ★ | - | ★ | ★★ | ★ | ★ | - | 7 |
| Yamamoto, 2016 | ★ | ★ | - | ★ | ★★ | - | ★ | - | 6 |
| Yu, 2019 | ★ | - | ★ | ★ | ★★ | - | ★ | - | 6 |

| | | | | | | | | | |
|---|---|---|---|---|---|---|---|---|---|
| Li, 2014 | ★ | ★ | - | ★ | ★★ | ★ | ★ | - | 7 |
| Liu, 2012 | ★ | ★ | - | ★ | ★★ | ★ | ★ | - | 7 |
| Yuan, 2011 | ★ | ★ | - | ★ | ★★ | ★ | ★ | - | 7 |
| Bruyère, 2010 | ★ | ★ | ★ | ★ | ★★ | - | ★ | - | 7 |
| Sáenz-López, 2013 | ★ | ★ | ★ | ★ | ★★ | - | ★ | - | 7 |
| Lu, 2015 | ★ | - | ★ | ★ | ★★ | - | ★ | - | 6 |
| Liu, 2020 | ★ | ★ | ★ | ★ | ★★ | - | ★ | - | 7 |
| Zhai, 2008 | ★ | ★ | - | ★ | ★★ | ★ | ★ | - | 7 |
| Li, 2010 | ★ | ★ | - | ★ | ★★ | ★ | ★ | - | 7 |
| Sivaprasad, 2013 | ★ | ★ | - | ★ | ★ | ★ | ★ | - | 6 |
| Cheng, 2014 | ★ | ★ | - | ★ | ★★ | ★ | ★ | - | 7 |
| Zhao, 2015 | ★ | ★ | - | ★ | ★★ | - | ★ | - | 6 |
| Bingül, 2016 | ★ | ★ | - | ★ | ★★ | ★ | ★ | - | 7 |
| Nie, 2016 | ★ | ★ | - | ★ | ★★ | - | ★ | - | 6 |
| Ai, 2020 | ★ | ★ | - | ★ | ★★ | ★ | ★ | - | 7 |

Criteria 1 = Adequate definition of case, Criteria 2 = Representativeness of the case, Criteria 3 = Selection of controls, Criteria 4 = Definition of controls, Criteria 5 = Control for important factor, Criteria 6 = Assessment of exposure, Criteria 7 = Same method of ascertainment for cases and controls, Criteria 8 = Non-Response rate.

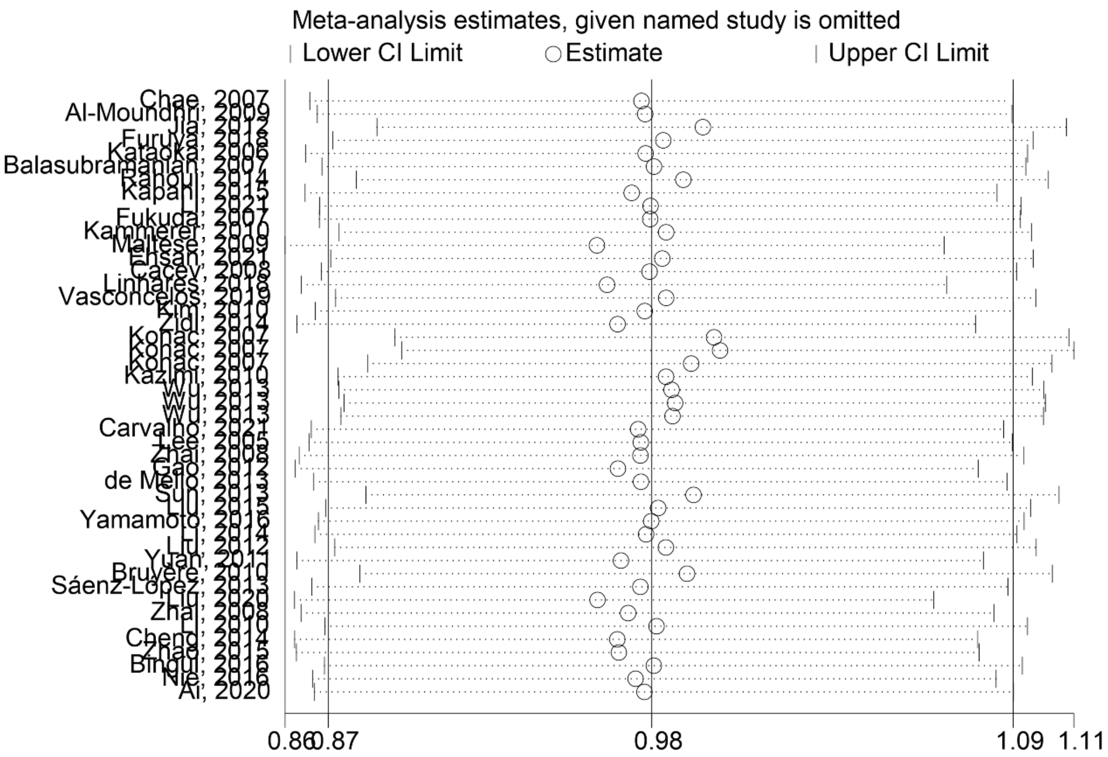


**Supplementary Figure S1.** Sensitivity analyses of the correlation of VEGF-460 with cancer risk in the dominant model (TC + CC vs TT).

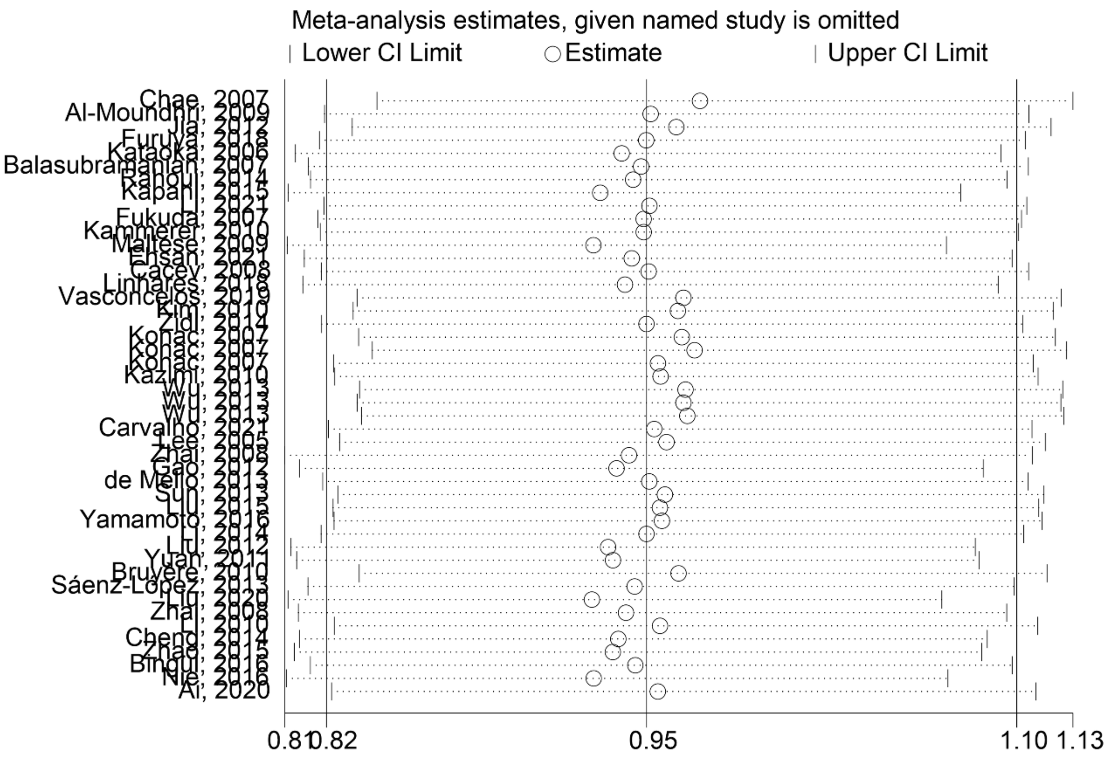


**Supplementary Figure S2.** Sensitivity analyses of the correlation of VEGF-460 with cancer risk in the recessive mode (CC vs TC + TT).

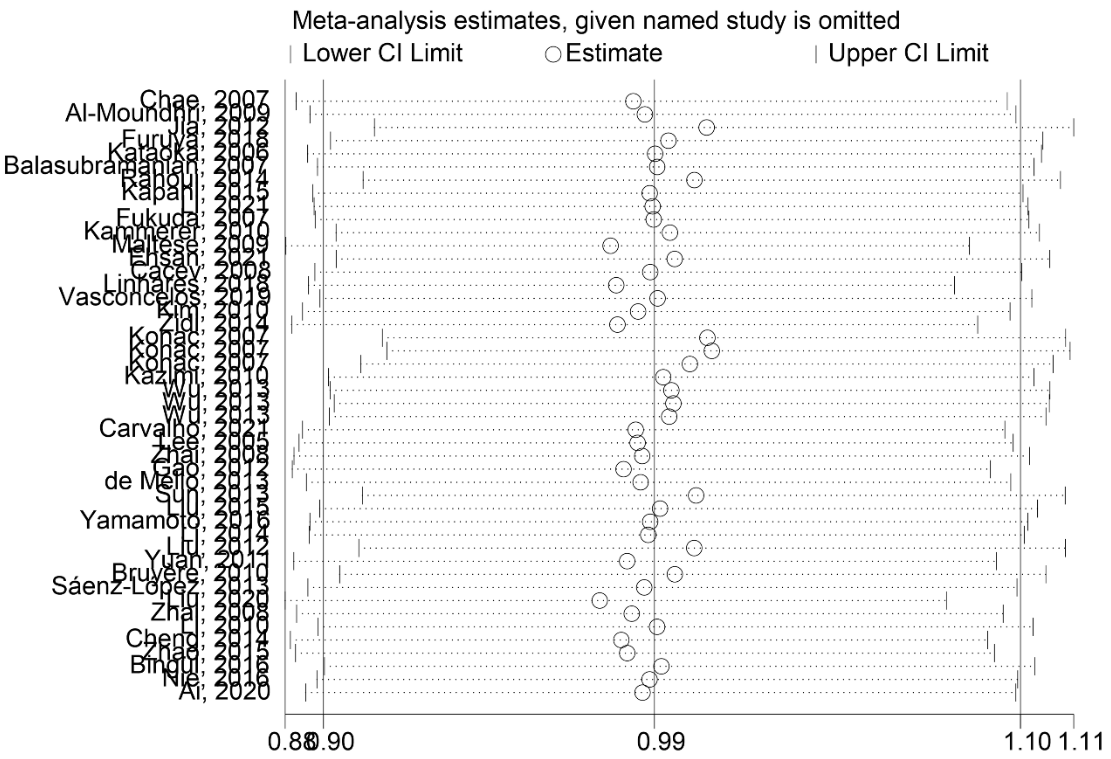


**Supplementary Figure S3.** Sensitivity analyses of the correlation of VEGF-460 with cancer risk in the heterozygous model (TC vs TT).

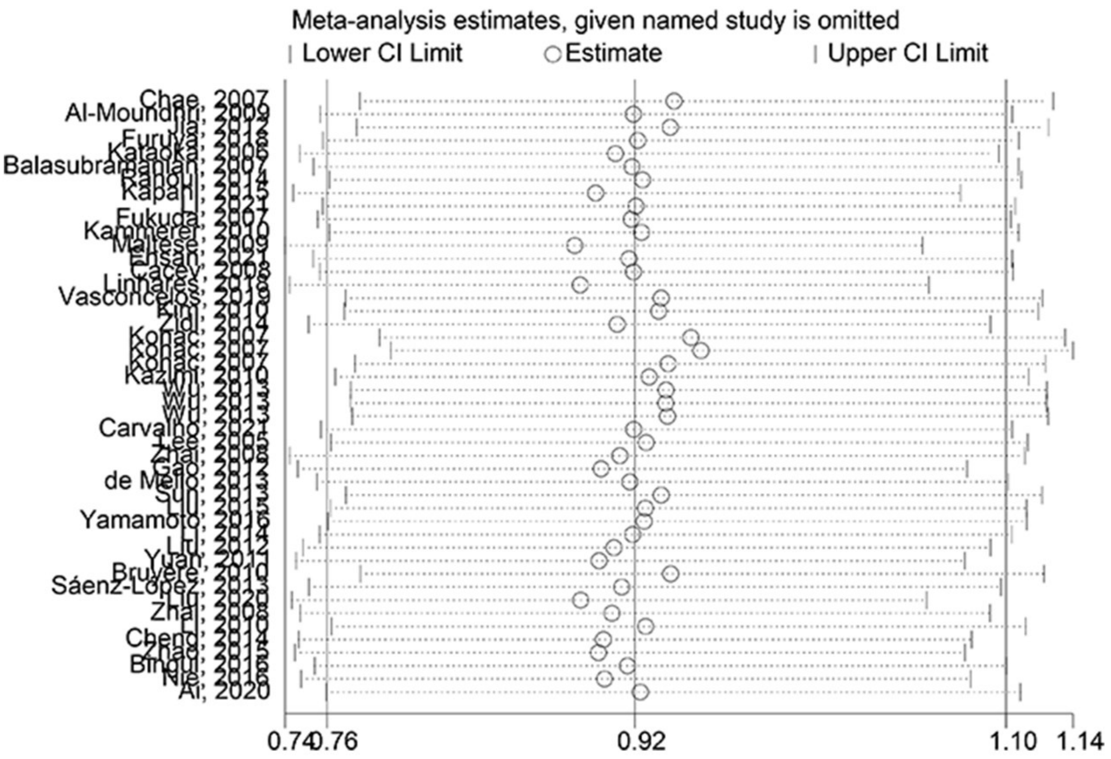


**Supplementary Figure S4.** Sensitivity analyses of the correlation of VEGF-460 with cancer risk in the homozygous model (CC vs TT).

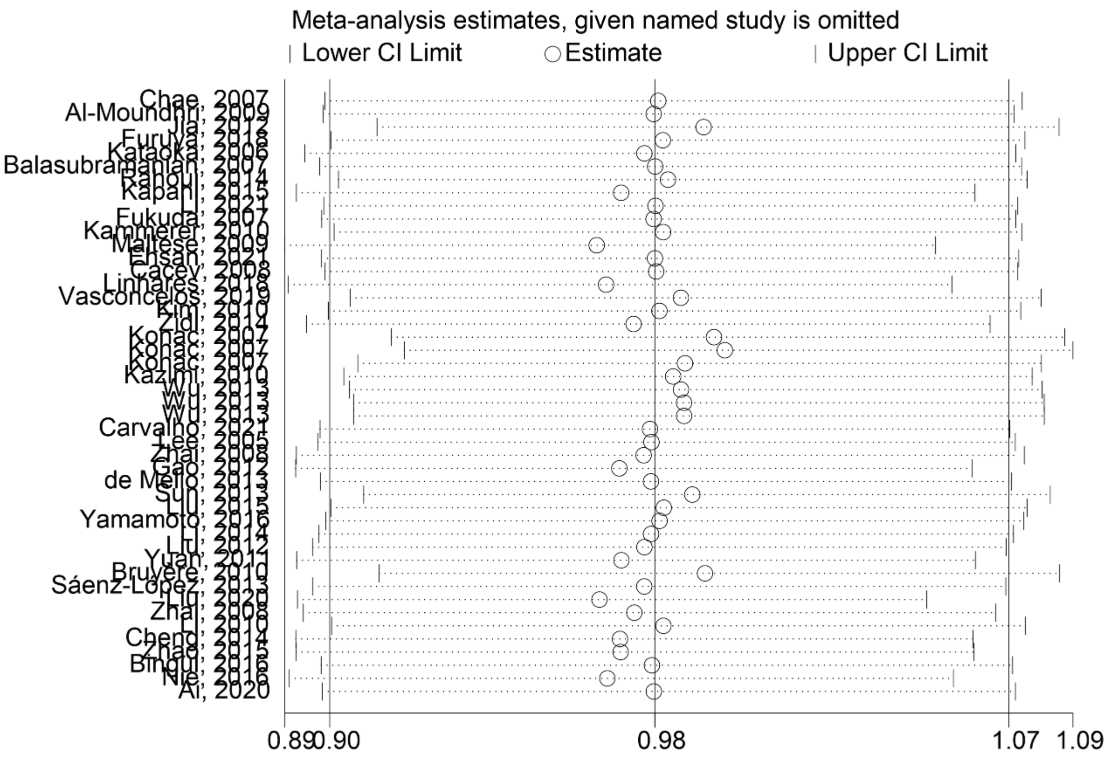


**Supplementary Figure S5.** Sensitivity analyses of the correlation of VEGF-460 with cancer risk in the additive model (C vs T).
