# Supplementary material for: The relationship between VEGF-460(T>C) polymorphism and cancer risk: A systematic review and meta-analysis based on 46 reports
Source: Medicine (Baltimore). 2023 Jun 30;102(26):e34089. doi: 10.1097/MD.0000000000034089 (PMC10313293; doi:10.1097/MD.0000000000034089)
Supplement: Supplementary file 2 [file medi-102-e34089-s002.pdf]

Supplemental Table 2. quality evaluation results in the included studies.

No	Author and Year	Selection				Comparability	Exposure			Score
		Criteria 1	Criteria 2	Criteria 3	Criteria 4	Criteria 5	Criteria 6	Criteria 7	Criteria 8	
1	Chae, 2007	★	★	★	★	★	-	★	-	6
2	Al-Moundhri, 2009	★	★	-	★	★★	★	★	-	7
3	Jia, 2012	★	★	-	★	★★	-	★	-	6
4	Furuya, 2018	★	★	★	★	★★	-	★	-	7
5	Kataoka, 2006	★	★	★	★	★★	-	★		7
6	Balasubramanian, 2007	★	★	-	★	★★	★	★	-	7
7	Rahoui, 2014	★	★	-	★	★★	-	★	-	6
8	Kapahi, 2015	★	★	-	★	★★	-	★	-	6
9	Maryam, 2016	★	★	★	★	★	-	★	-	6
10	Albalawi, 2020	★	-	★	★	★	-	★	-	5
11	Li, 2021	★	★	-	★	★★	★	★	-	7
12	Lin, 2003	★	-	-	★	★★	-	★	-	5
13	Fukuda, 2007	★	★	-	★	★★	-	★	-	6
14	Onen, 2007	★	-	-	★	★★	-	★	-	5
15	Li, 2017	★	-	-	★	★	-	★	-	4
16	Ku, 2005	★	★	-	★	★	★	★	-	6
17	Kammerer, 2010	★	★	-	★	★★	★	★	-	7
18	Borase, 2015	★	-	-	★	★★	-	★	-	5
19	Maltese, 2009	★	★	-	★	★★	-	★	-	6
20	Dassoulas, 2009	★	-	-	★	★★	-	★	-	5
21	Ehsan, 2021	★	★	-	★	★★	-	★	-	6
22	Cacev, 2008	★	★	★	★	★★	-	★	-	7
23	Jannuzzi, 2015	★	-	-	★	★★	-	★	-	5
24	Linhares, 2018	★	★	-	★	★★	★	★	-	7
25	Vasconcelos, 2019	★	★	★	★	★	★	★	-	7
26	Kim, 2010	★	★	-	★	★★	★	★	-	7
27	Zidi, 2014	★	★	-	★	★★	-	★	-	6
28	Konac, 2007	★	★	-	★	★★	-	★	-	6
29	Kazimi, 2010	★	★	-	★	★★	-	★	-	6
30	Wu, 2013	★	★	-	-	★★	★	★	-	6
31	Carvalho, 2021	★	★	-	★	★★	-	★	-	6
32	Lee, 2005	★	★	-	★	★★	★	★	-	7
33	Zhai, 2008	★	★	-	★	★★	-	★	-	6
34	Gao, 2012	★	★	-	★	★★	-	★	-	6
35	de Mello, 2013	★	★	-	★	★★	-	★	-	6
36	Sun, 2013	★	★	-	★	★★	★	★	-	7
37	Liu, 2015	★	★	-	★	★★	★	★	-	7
38	Yamamoto, 2016	★	★	-	★	★★	-	★	-	6
39	Yu, 2019	★	-	★	★	★★	-	★	-	6
40	Li, 2014	★	★	-	★	★★	★	★	-	7

41	Liu, 2012	★	★	-	★	★★	★	★	-	7
42	Yuan, 2011	★	★	-	★	★★	★	★	-	7
43	Bruyère, 2010	★	★	★	★	★★	-	★	-	7
44	Sáenz-López, 2013	★	★	★	★	★★	-	★	-	7
45	Lu, 2015	★	-	★	★	★★	-	★	-	6
46	Liu, 2020	★	★	★	★	★★	-	★	-	7
47	Zhai, 2008	★	★	-	★	★★	★	★	-	7
48	Li, 2010	★	★	-	★	★★	★	★	-	7
49	Sivaprasad, 2013	★	★	-	★	★	★	★	-	6
50	Cheng, 2014	★	★	-	★	★★	★	★	-	7
51	Zhao, 2015	★	★	-	★	★★	-	★	-	6
52	Bingül, 2016	★	★	-	★	★★	★	★	-	7
53	Nie, 2016	★	★	-	★	★★	-	★	-	6
54	Ai, 2020	★	★	-	★	★★	★	★	-	7

Criteria 1 = Adequate definition of case, Criteria 2 = Representativeness of the case, Criteria 3 = Selection of controls, Criteria 4 = Definition of controls, Criteria 5 = Control for important factor, Criteria 6 = Assessment of exposure, Criteria 7 = Same method of ascertainment for cases and controls, Criteria 8 = Non-Response rate.
