# Supplementary material for: The relationship between VEGF-460(T>C) polymorphism and cancer risk: A systematic review and meta-analysis based on 46 reports
Source: Medicine (Baltimore). 2023 Jun 30;102(26):e34089. doi: 10.1097/MD.0000000000034089 (PMC10313293; doi:10.1097/MD.0000000000034089)

Supplemental Figure 1. Supplemental digital content. Figure that set out the results of sensitivity analysis. (A: dominant model; B: Recessive model; C: Homozygous model; D: heterogeneous model; E: additive model.)

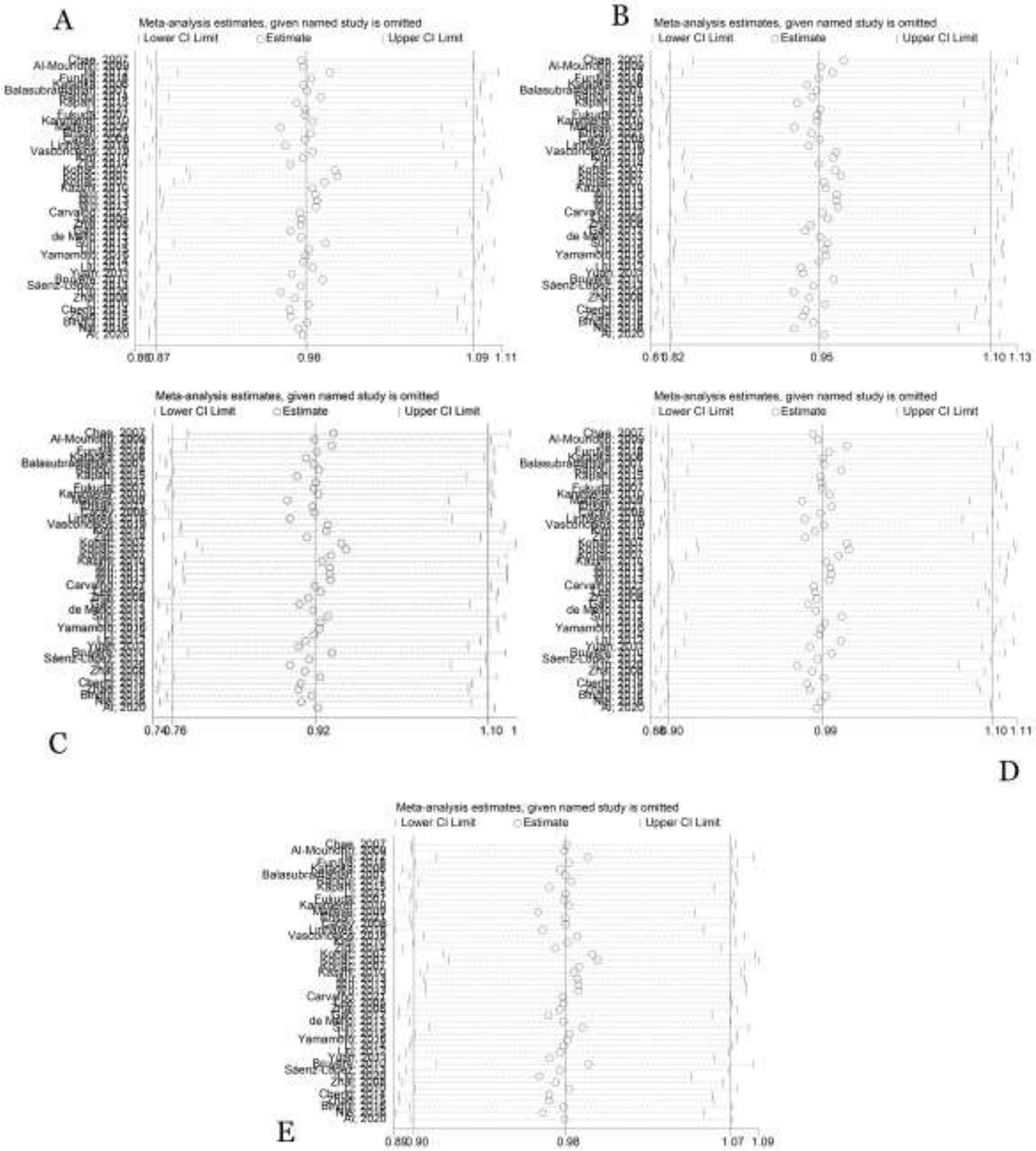

Supplement: Supplementary file 3 [file medi-102-e34089-s003.pdf]
